# He Cracked Malaria’s Genome in Mali. Now He’s Advising the UN

**DOI:** 10.1021/acscentsci.6c01035

**Published:** 2026-06-26

**Authors:** Scovian Lillian

## Abstract

Abdoulaye Djimdé spent decades decoding malaria drug resistance from inside the communities where it strikes. Now, in addition to this fieldwork, he’s advising on global science policy.

## Graphical abstract

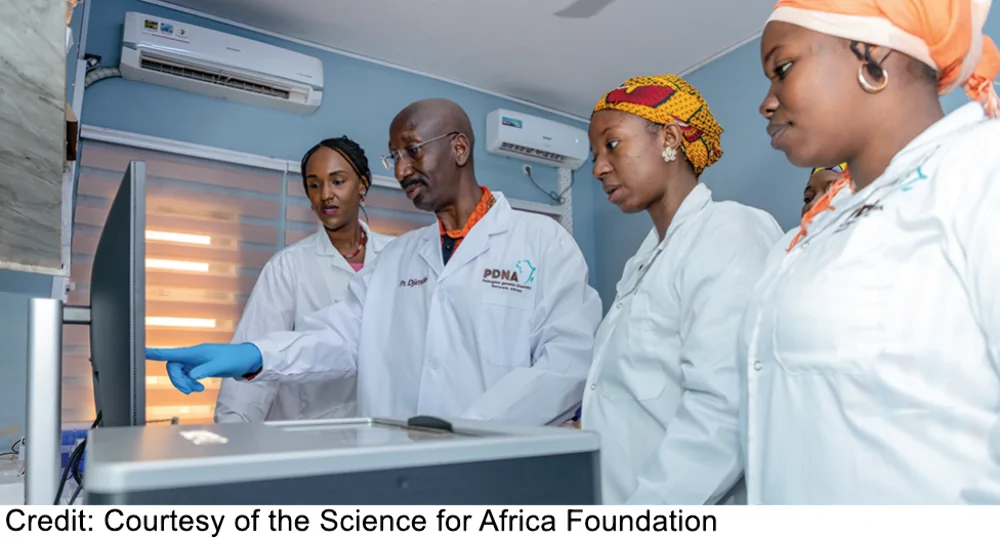

When Abdoulaye Djimdé finally had a moment to talk, he was in a hotel room in Nairobi, Kenya, his Internet connection flickering, cameras off to save bandwidth. He had another meeting to get to. He always has another meeting to get to.

**Figure:**
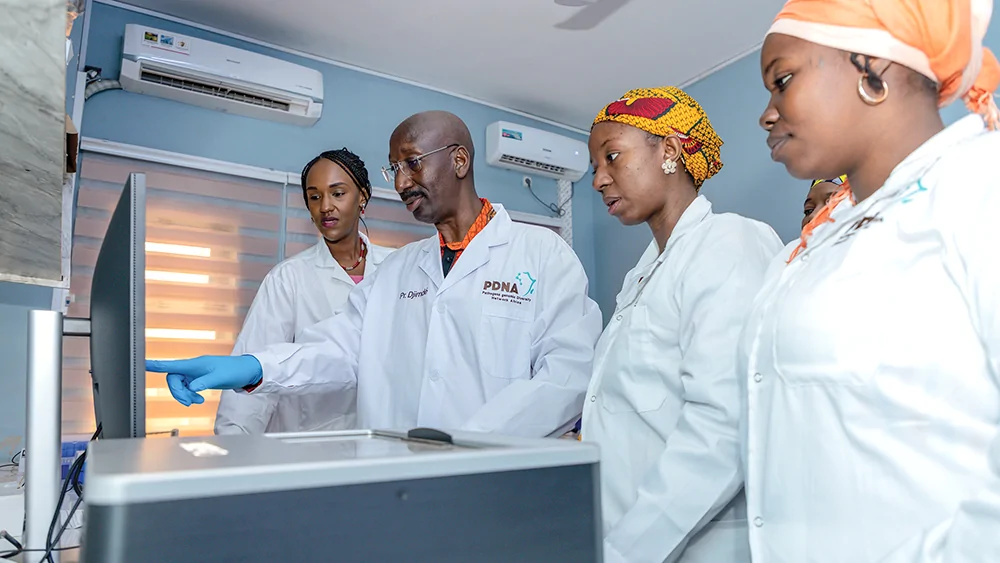
Abdoulaye Djimdé (second from left) reviews genomic data with researchers Fatoumata Maiga (from left), Hinda Doucoure, and Maimouna Dembele at the Malaria Research and Training Center at the University of Sciences, Techniques, and Technologies of Bamako. Credit: Courtesy of the Science for Africa Foundation.

Djimdé was appointed in March to the United Nations Secretary-General’s Scientific Advisory Board, a body that advises on science and technology. It is, he says, a space where African scientific voices are needed.

The board provides independent, evidence-based guidance on science and technology for sustainable development and policymaking. Djimdé’s appointment brings an African viewpoint, particularly on infectious diseases, genomic surveillance, and research capacity.

“We African scientists research malaria because we live the problem. It is a very important advisory role—the committee tackles important science issues in terms of innovation, directions that the world’s science is taking, and discusses how these new directions and innovations would impact society at large,” Djimdé says. “Having African voices represented in those discussions would bring into consideration the perspective of the African continent.”

That perspective has been forged over decades of fieldwork. As a professor of parasitology and mycology at the University of Sciences, Techniques, and Technologies of Bamako—in Mali, one of the world’s most malaria-burdened countries—Djimdé and his lab sit at the intersection of field science and global health policy. His team tests drug candidates against parasites collected directly from patients rather than strains that have spent decades adapting to laboratory conditions.

“In the Global North, colleagues tend to work with what I call pet parasites—parasites that have been in the lab for 30 years, adapted to lab conditions, grown and regrown so many times that their features are sometimes different from what you find in patients today,” Djimdé says.

Working with current field isolates, his team reaches conclusions about drug candidates that diverge sharply from findings made in laboratories that use only laboratory models. For example, a study Djimdé’s lab published recently compares how well 14 antimalarial drugs performed against *Plasmodium falciparum*, the deadliest malaria parasite spread by mosquitoes. The team noticed significant differences in drug responses and linked those to each of three sites the parasites had been collected from. The variability the researchers found in drug resistance could inform treatment strategies in each region, the researchers report.

That focus on field research had defined Djimdé’s work from the beginning. His doctoral research at the University of Maryland, Baltimore, led him to investigate why chloroquine, the frontline drug against malaria for decades, had stopped working in sub-Saharan Africa starting in the 1970s. In the 1990s, he and colleagues tested how effective chloroquine was in treating uncomplicated cases of malaria in rural Mali.

The root cause of the resistance lay in a gene—not one in humans but in the parasite’s DNA. Between 1996 and 2001, he worked as a visiting fellow with scientists at the National Institutes of Health (NIH) to identify a mutation in one of the parasite’s genes, a section of DNA that codes for the protein *Plasmodium falciparum* chloroquine-resistant transporter, or PfCRT. The NIH team found that the mutated protein stopped chloroquine from entering the parasite’s vacuole, where the drug would otherwise kill the bug.

Djimdé’s team then validated the mutation in the field in Mali, treating malaria patients with chloroquine and screening surviving parasites for the mutation. Every resistant parasite carried it; every susceptible one did not. “That was really the first validation of this point mutation in the field,” Djimdé says.

The group then developed an assay that required only a dried blood spot on filter paper—no cold chain or specialist equipment needed—and that could be mailed like a normal letter. The mutated gene became the most widely used molecular tool for tracking chloroquine resistance globally.

The approach of taking a complex scientific finding and making it work in the field with minimal resources is what Djimdé brings to the UN advisory board, where he argues that scientific innovations must be judged by whether they can reach the people who need them most.

By the early 2000s, large international genomics consortia were generating population-level data on *Plasmodium falciparum* from across the world. A conclusion kept surfacing: African parasite populations showed low genetic diversity. Compared with parasites from Asia or Latin America, African parasites appeared uniform. In the language of those meetings, they were the negative control—scientifically uninteresting.

“We used to attend these meetings and think, That is not the full story. We knew that malaria behaved differently in different parts of the continent,” Djimdé says. “And we knew that must have been driven by genetic differences.”

In 2013, Djimdé and a group of other African scientists formally launched the Plasmodium Diversity Network Africa, now called the Pathogen Genomic Diversity Network Africa. The group eventually assembled and analyzed parasite genes—all collected from the field—from 15 African countries.

The results overturned the consensus. Parasites from southern, central, eastern, and western Africa were all genetically distinct from each other. “It completely changed the way the field thought about and studied African parasites,” Djimdé says.

He had to create an entirely Africa-led research network just to ask the right questions, something Djimdé says informs his outlook as he begins work with the UN. Science governance, he argues, has the same problem genomics had: the frameworks are built without African input, and the blind spots follow.

While building the network, Djimdé became involved in one of the most consequential problems in malaria drug development. Pyramax, a combination drug, had shown strong efficacy even against resistant strains of malaria, but early trials flagged liver toxicity cases. As part of its approval of Pyramax in 2012, the European Medicines Agency restricted it to a single lifetime dose, pending more data.

For a continent where children suffer several malaria episodes per year, that was effectively a disqualification. “A drug that you can only use once in a lifetime is not a drug for Africa,” Djimdé says.

Between 2011 and 2016, his team tracked patients across repeated infections, working with a West African clinical trials network, Pyramax’s South Korean codeveloper, and the nonprofit and drug codeveloper Medicines for Malaria Venture. Each patient was followed for 2 years, a novel design at the time.

After tracking more than 14,000 malaria episodes, the researchers showed that Pyramax was both safe and effective with repeated use. The drug is now registered in more than 20 countries across sub-Saharan Africa.

This type of collaboration is an example of what’s possible, Djimdé says. “When you match our local, life-driven perspective with the technology and know-how of our Northern colleagues, you find more useful solutions.”

*Scovian Lillian is a freelance contributor to* Chemical & Engineering News, *an independent news publication of the American Chemical Society.*

